# Quantitative profiling of spreading-coupled protein tyrosine phosphorylation in migratory cells

**DOI:** 10.1038/srep31811

**Published:** 2016-08-24

**Authors:** Yajun Xie, Jinlong Wang, Yuanya Zhang, Xiaofei Liu, Xiaorong Wang, Kehui Liu, Xiahe Huang, Yingchun Wang

**Affiliations:** 1 State Key Laboratory of Molecular Developmental Biology, Institute of Genetics and Developmental Biology, Chinese Academy of Sciences, No.1 West Beichen Rd., Beijing 100101, China; 2 University of the Chinese Academy of Sciences, Beijing 100049, China

## Abstract

Protein tyrosine phosphorylation is an important mechanism that regulates cytoskeleton reorganization and cell spreading of migratory cells. A number of cytoskeletal proteins are known to be tyrosine phosphorylated (pY) in different cellular processes. However, the profile of pY proteins during different stages of cell spreading has not been available. Using immunoafffinity enrichment of pY proteins coupled with label free quantitative proteomics, we quantitatively identified 447 pY proteins in the migratory ECV-304 cells at the early spreading (adhesion) and the active spreading stages. We found that pY levels of the majority of the quantified proteins were significantly increased in the active spreading stage compared with the early spreading stage, suggesting that active cell spreading is concomitant with extra tyrosine phosphorylation. The major categories of proteins impacted by tyrosine phosphorylation are involved in cytoskeleton and focal adhesion regulation, protein translation and degradation. Our findings, for the first time, dissect the cell spreading-specific pY signals from the adhesion induced pY signals, and provide a valuable resource for the future mechanistic research regarding the regulation of cell spreading.

Integrin-mediated cell adhesion to and spreading on extracellular matrix (ECM) is critical for cell survival and migration^1^. When cell surface integrin receptors are activated by the engagement of the ligand matrix proteins such as fibronectin, signal transduction cascade will be initiated by integrin activation leading to the subsequent activation of Rho family small GTPases such as RhoA, Rac, and CDC42^2^. The activated small GTPases in turn regulate cell spreading through coordinated regulation of actin cytoskeleton reorganization and focal adhesions formation and turnover^3^. Because of its functional significance in both physiological and pathological processes, tremendous amount of effort in investigating the mechanism controlling this process has been made. Unfortunately, the detailed mechanism underlying this process is still elusive.

Active cell spreading requires dynamic actin cytoskeleton reorganization and focal adhesion formation and turnover, both processes are known to be regulated by tyrosine phosphorylation^4^,^5^. For example, the autophosphorylation of FAK Y397 upon integrin activation can activate FAK by providing a docking site for the cytoplasmic kinase Src^6^. Src binding and phosphorylation of FAK Y576/577 leads to full activation of FAK, which plays a critical role in regulating cell spreading and migration through regulating formation and turnover of focal adhesions^7^. Paxillin is the other well-known pY protein, and the functional significance of its phosphorylation at Y31 and Y118 in focal adhesion regulation has been well studied^8^. P130Cas is also an important pY protein in regulating cell adhesion and spreading. The Y249 phosphorylation of p130Cas can recruit Crk to form a protein complex, which serves as the molecular switch to control cell spreading and cell migration^9^. Because of the functional importance of the phosphorylation-regulated activities of adhesion related proteins, accumulating effort has been recently focused on the large-scale identification of adhesion machinery proteins as well as their phosphorylation status^10^,^11^,^12^. Unfortunately, tyrosine phosphorylation is underrepresented in the catalogue of these reported adhesion-related phosphoproteins as it is generally much less abundant than serine- or threonine-phosphorylation^13^. Moreover, different stages of cell spreading such as the early and the active spreading stages may require different activities conferred by the differential phosphorylation of proteins, and the dynamic changes of the phosphorylation status of pY proteins at the different spreading stages have not been specifically documented, at least at a global level. Lacking of this information has seriously impeded the paces towards the understanding of the mechanism regulating cell adhesion and spreading.

Here, using ECV-304 cell line, we determined the time points when suspended cells reach the states of fully attached and active spreading on fibronectin-coated dishes, and then quantitatively compared the pY proteomes of the cells in the two states. We found that overall more proteins have increased tyrosine phosphorylation in active spreading cells than in fully attached cells. We bioinformatically analyzed the functional significance of the pY proteins differentially phosphorylated in the two stages. Our results should serve as a useful resource for the future mechanistic studies of the regulation of cell adhesion and spreading.

## Results

### Proteins are differentially tyrosine phosphorylated between the early and the active spreading stages of the migratory cells

**Figure 1:**
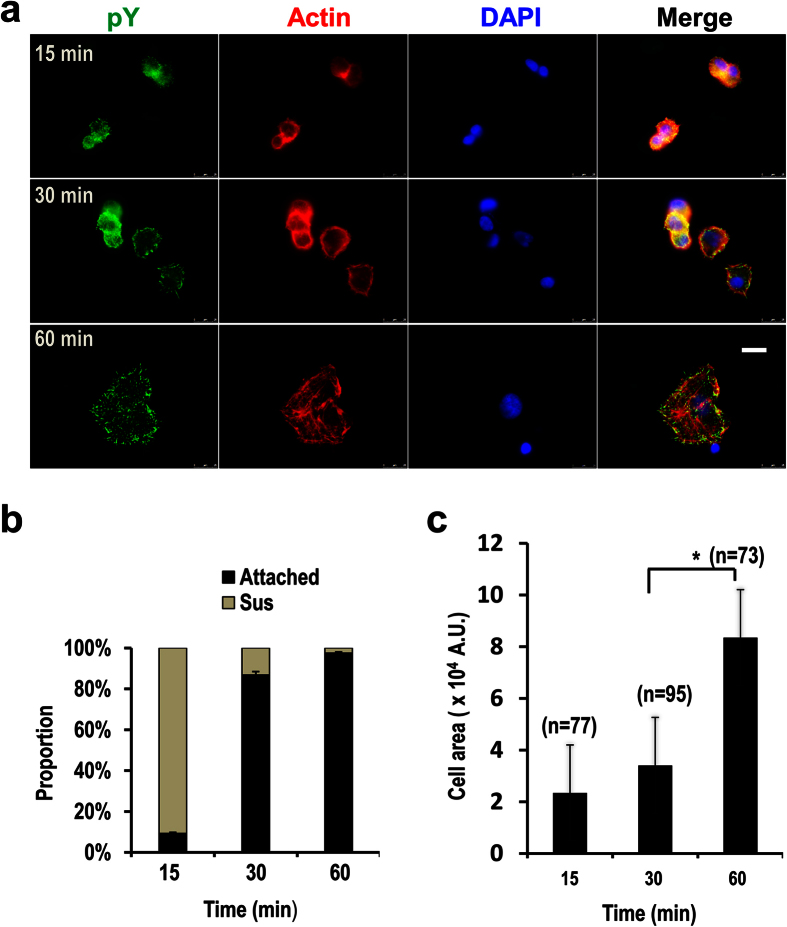
Determination of the early and the active spreading stages of migratory cells. **(a)** ECV-304 cells were seeded on 5 μg/ml fibronectin coated cover glass and allowed to attach and spread. The cells were fixed at indicated time points and stained with anti-pY antibody, Alexa 546 phalloidin (for actin), and DAPI (for nuclei). Scale bar: 25 μm. **(b)** The proportions of the attached cells (Attached) versus the floating cells (Sus) at each time point as indicated during the time course of cell attaching and spreading. The error bars represent standard deviations. (**c)** The areas of attached cells in (**a**) were quantified using MetaMorph. The significance of difference was determined by non-paired Student’s *t*-test. The number of cells used for quantifying the areas at each time point is shown above each bar. Error bars: standard deviations. *p < 0.05.

**Figure 2:**
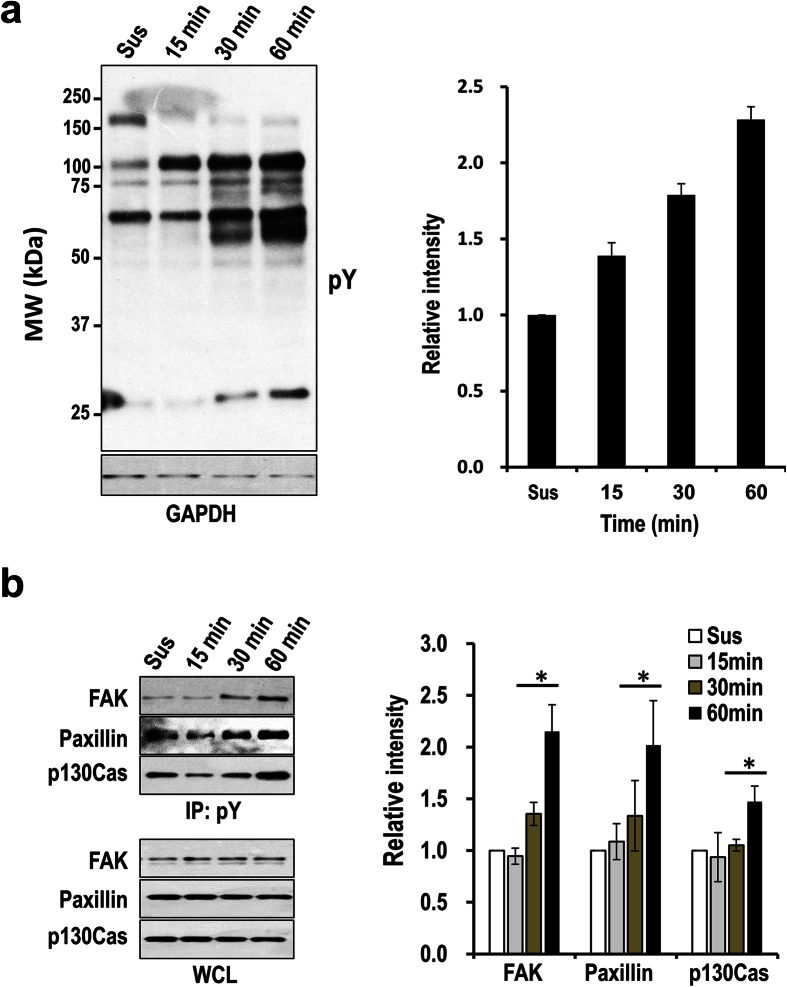
Differential protein tyrosine phosphorylation in cells at different spreading stages. (**a**) Western-blotting detection of the total pY levels from the cells seeded on 5 μg/ml fibronectin coated dishes for the indicated times. Cells in suspension (Sus) were used as the control, and GAPDH was probed as the loading control (Left panel). Shown is the representative result from three independent experiments. The Western-blotting results were quantified with densitometry and shown in the bar graph (Right panel). The combined pixel intensity from all detectable bands in each lane was used for the analysis. The relative intensities at the different time points were shown as the fold changes over that of the cells in suspension. Error bars indicate standard deviations. (**b)** Detection of total pY level of FAK, Paxillin, and p130Cas in the cells treated as in **(a)** using immunoprecipitation coupled with Western-blotting. The total pY proteins were immunoprecipitated from the WCL, and the pY form of FAK, Paxillin, and p130Cas were detected by Western-blotting using antibodies specific for these proteins. Total levels of the indicated proteins in WCL were also probed as the loading control (Left panel). The pY forms of the indicated proteins detected by immunoprecipitation and Western-blotting were quantified with densitometry from three independent experiments and shown in the bar graph (Right panel). Error bars indicate standard deviations. *p < 0.05. Note that phosphorylation of Paxillin is slightly higher in control cells (Sus) than the cells at the time point 15 min, this is probably caused by cell manipulation such as buffer changes during the experiment.

To determine the time points representing the early and the active spreading stages, we performed the spreading assay using ECV-304, a cell line derived from human umbilical vein endothelial cells and has been used as a model for angiogenesis study^14^. The cell line expresses integrin receptors for fibronectin, laminin, and collagen, and can migrate fast on these extracellular matrix proteins^14^. The cells were seeded on 5 μg/ml fibronectin-coated dishes, and the majority of them started to but did not firmly attach at 15 min. The firm attachment was achieved at 30 min (Fig. 1a,b), as indicated by the observation of just a few floating cells when slightly shaking the dishes, which is in sharp contrast to the cells at 15 min when the majority of cells are still able to float if shaking the dishes. The firmly attached cells at 30 min displayed increased number of adhesion sites compared with those at 15 min as observed by immunofluorescence staining. However, the sizes of the cells at 30 min, which are indicated by cell areas, were not significantly increased compared with those at 15 min (Fig. 1c), suggesting that at this time point cell spreading did not start or was at the very early stage if the process has already been initiated. The cells started to spread after this stage and reached an active spreading stage at 60 min as characterized by increased cell sizes and the number and the size of adhesion sites (Fig. 1a,c). The overall pY signal, as measured by the immunofluorescence staining and Western-blotting, is also increased at 60 min (Figs 1 and 2). The increase of pY is a strong indicator of their functional importance in the regulation of cell spreading. Thus, it is necessary to identify the pY proteins before addressing their functional significance.

### Large-scale quantitative identification of pY proteins at the early and the active spreading stages

**Figure 3:**
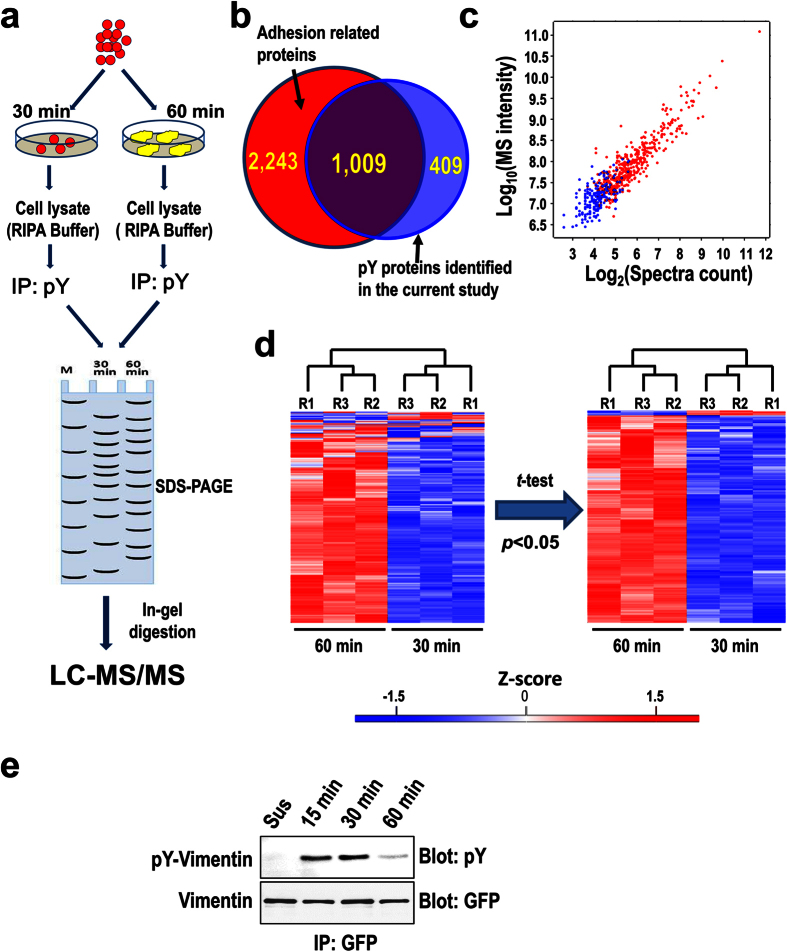
Quantitative proteomic analysis of pY proteins in the early and the active stages of migratory cells. **(a)** Schematic representation of the strategy for the proteomic analysis of the pY proteins. (**b)** The Venn diagram shows the comparison of the pY proteins identified in the current study with the adhesion related proteins identified by Schiller H. *et al*.^12^, The numbers of uniquely-identified and overlapping proteins from both studies are shown in the diagram. **(c)** The 2-D scatter-plot shows the spectral counts (x-axis) and MS intensities (y-axis) for all proteins containing quantitative information for all three replicates in at least one time point. The blue dots represent proteins containing quantitative information for all three replicates at only one time point, the red dots represent proteins with quantitative information for all three replicates at both time points. (**d)** Evaluation of the reproducibility of the quantitation using clustering analysis. The Z-scores of the normalized MS intensities of the 447 proteins with sufficient quantitative information (as indicated by the red dots in panel c) are color-coded to show the relative protein abundance (red: high, blue: low), and hierarchical clustering analysis was performed using the software Perseus with default parameters. Student’s *t*-test was used to filter out the proteins quantitated with low reproducibility (*p* < 0.05), which reduced the number of proteins from 447 to 371. The scale bar indicates the Z-scored values of MS intensities. (**e**) Validation of downregulation of pY-Vimentin at 60 min by immunoprecipitation and Western-blotting. The cells expressing GFP-Vimentin were allowed to adhere on 5 μg/ml fibronectin for the indicated times or kept in suspension (Sus) as indicated, the cells were then harvested and lysed. The fusion protein was immunoprecipitated from the WCLs using anti-GFP antibody, the pY level of immunoprecipitated GFP-Vimentin was detected by Western-blotting using anti-pY antibody, and the total level of GFP-Vimentin was detected by Western-blotting using anti-GFP antibody.

PY proteins are generally low abundant and thus require specific enrichment to ensure effective proteomic analysis. The overall strategy of the quantitative proteomic analysis is illustrated in Fig. 3a. Briefly, the cells attached on fibronectin were lysed at the early and the active spreading stages, which are represented by the time points 30 min and 60 min, respectively. The pY proteins were enriched from each whole cell lysate (WCL) by immunoaffinity purification. The enriched pY proteins were separated with SDS-PAGE and digested in-gel to generate tryptic peptides for liquid chromatography coupled with tandem mass spectrometry (LC-MS/MS) analysis. For label free quantification, three biological replicates were included for the analysis.

In total, 1,418 proteins that are potentially tyrosine phosphorylated were identified (Supplemental Table 1). Comparing these proteins with one of the largest datasets of recently identified adhesion related proteins reveals that 1,009 proteins are overlapping (Fig. 3b and Supplemental Table 1)^12^, suggesting that the majority of proteins identified in the current study (71.2%) are directly or indirectly involved in cell adhesion and/or spreading. Indeed, 50 proteins identified in the current study were previously defined as the adhesome components, which constitute 32% of all defined adhesome components (Supplemental Table 1)^15^. Among all identified proteins 447 proteins contain quantitative information (MS intensity) for all three replicates in both stages and 205 proteins have quantitative information for all three replicates in only one stage, either for the point 30 min or 60 min. Ranking the proteins by their abundance as indicated from their spectra counts and MS intensities revealed that the 205 proteins are exclusively low abundance (Fig. 3c). These low abundance proteins were excluded and the 447 proteins with better quantitative information were kept for further analysis to improve the confidence and accuracy of the quantitation (Supplemental Table 2). It is of note that all these proteins contain 2 or more identified peptides, which warrants the high quality of the qualitative and the quantitative proteomic analyses. Remarkably, high percentage (84.1%) of the proteins were estimated to be true pY proteins by comparing them with the previously identified pY proteins in the PhosphoSitePlus^16^, the largest phosphorylation site database.

The high reproducibility of the label free quantitation was demonstrated by the Hierarchical clustering analysis of the normalized MS intensities, which correctly clustered the three replicates from the early and the active spreading stages together, respectively (Fig. 3d). To further improve the confidence of the quantitation, we only considered the proteins who passed the *t*-test (*p* < 0.05) and has a fold change higher than 1.5 as significantly changed between the two stages. This resulted in 317 significantly upregulated and 2 downregulated pY proteins in the active spreading stage (Supplemental Table 2). The result is consistent with the results of the immunofluorescence imaging and the Western-blotting (Figs 1, 2 and 3e), further confirming the general upregulation of tyrosine phosphorylation during the transition from the time points 30 min to 60 min. Remarkably, the majority of the significantly changed pY proteins (85%) were previously identified to be related with cell adhesion (Supplemental Table 2)^12^, which underscores the importance of the regulated tyrosine phosphorylation in cell spreading.

### Tyrosine phosphorylation significantly impacts cytoskeletal and focal adhesion proteins in the spreading process

**Figure 4:**
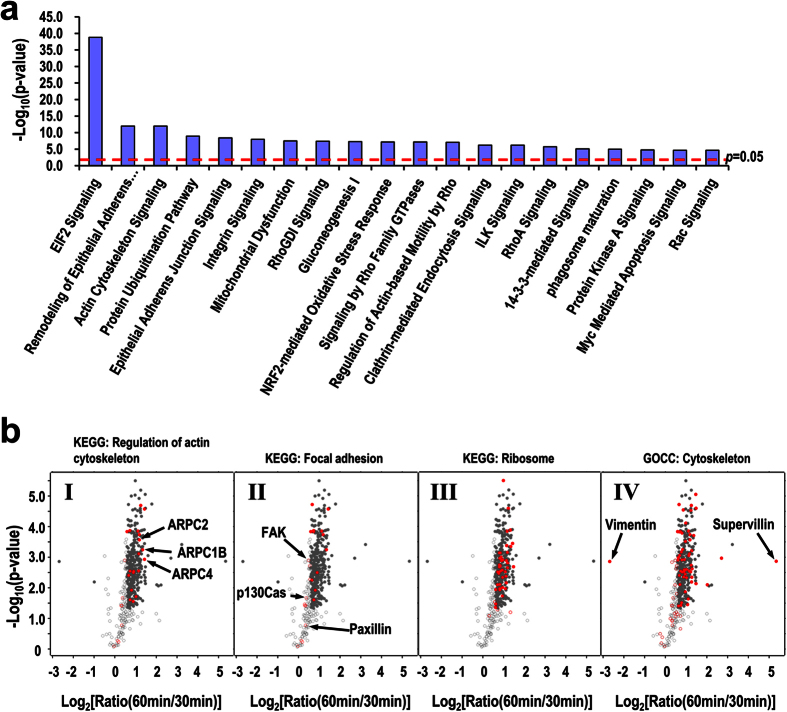
Bioinformatic analysis of the significantly changed pY proteins. (**a)** Representative pathways enriched in the significantly changed pY proteins as predicted by IPA analysis. The red dashed line indicates the threshold of the Fisher’s exact test of the significance (*p* < 0.05). (**b)** Scatter-plots showing the distribution of fold changes of pY proteins between the active and the early spreading stages. The red spots indicate the proteins involved in specific KEGG pathways or GO terms as indicated. X-axis: logarithm transformed fold change, Y-axis: logarithm transformed *p*-vlaue of Student *t*-test. The filled cycles represent proteins with a fold change >1.5 and a *p* < 0.05.

To reveal the potential functions of the identified pY proteins, we analyzed the canonical pathways significantly represented by these proteins using Ingenuity Pathway Analysis (IPA)^4^. The IPA analysis revealed that the majority of the top-scored canonical pathways are related with the regulation of actin cytoskeleton and focal adhesions, including remodeling of epithelial adherens junctions, actin cytoskeleton signaling, integrin signaling, RhoA signaling, Rac signaling, and etc. (Fig. 4a and Supplemental Table 3). Tyrosine phosphorylation of many proteins in these pathways is important in regulation of cytoskeleton assembly, focal adhesion formation and turnover, pseudopodia protrusion, and cell migration^4^,^5^,^7^,^17^. KEGG pathway analysis also mapped 27 and 23 proteins to the pathways regulation of actin cytoskeleton and focal adhesion, respectively (Fig. 4b, panels I and II). Gene ontology (GO) analysis revealed that 76 proteins are associated with the term cytoskeleton (GOCC: cellular component), including 53 proteins that are significantly upregulated in the active spreading stage (Fig. 4b, panel IV). Taken together, these observations suggest that cytoskeletal and focal adhesion protein is one of the major categories of proteins with upregulated tyrosine phosphorylation from the early to the active spreading stages.

Notably, the EIF2 signaling pathway mainly composed of ribosomal proteins is the most significantly enriched in the upregulated pY proteins (Fig. 4a,b). The majority of the ribosomal proteins contain previously identified pY sites in the PhosphositePlus database, though their functional significance remains elusive. It is conceivable to presume that the tyrosine phosphorylation of ribosome and a few translational initiation factors is necessary for optimal translation activity in supplying new functional proteins to support the dynamic cell spreading. In line with this, protein ubiquitination pathway is also one of the top-scored pathways enriched in the upregulated pY proteins (Fig. 4a), suggesting that the elevated protein degradation activity may also be required to remove non- or mal-functional proteins in time to meet the fast and diverse structural and signaling changes during the process.

### Identification of specific pY sites

**Figure 5:**
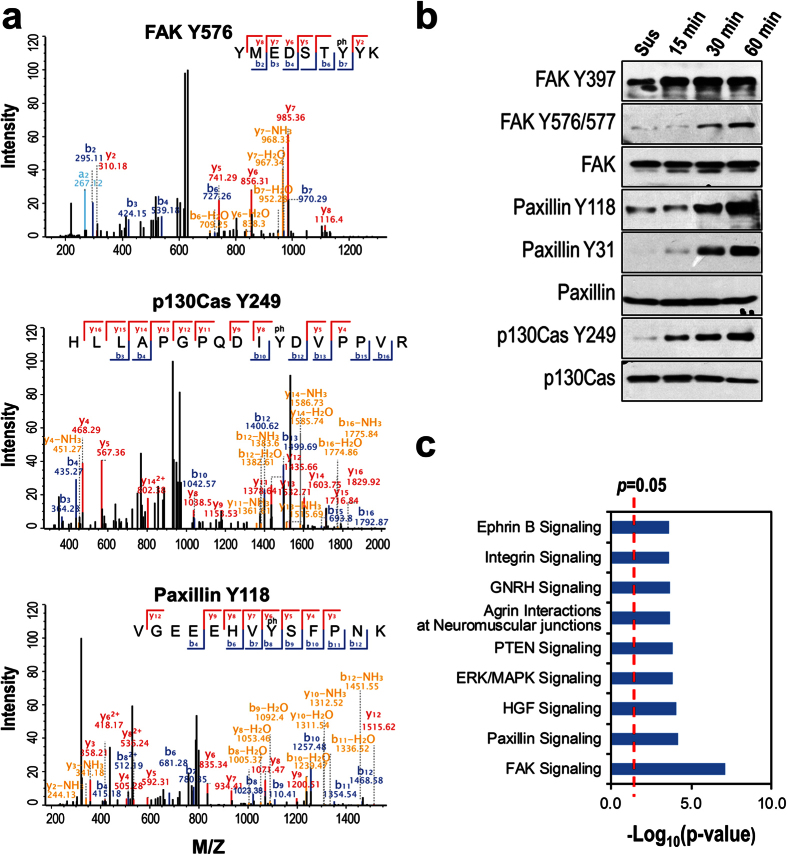
Identification of pY sites. (**a)** The spectra show the identification of specific pY sites on FAK, p130Cas, and paxillin. (**b)** Western-blotting detection of the specific pY sites in the two spreading stages, the corresponding proteins were also probed as the loading control. (**c)** Canonical pathways enriched in the proteins with identified pY sites as predicted by IPA analysis. X-axis: the logarithm-transformed *p*-value.

A total of 118 specific pY sites from 99 proteins were identified by the database search using tyrosine phosphorylation as one of the variable modifications (Supplemental Table 4). Many of the pY sites are known to play critical roles in regulation of cytoskeleton and focal adhesion such as FAK Y576, p130CAS Y249, and Paxillin Y118^8^,^9^,^18^ (Fig. 5a). Though our proteomic analysis did not quantitatively analyze the pY sites, we examined the phosphorylation levels of a selected list of pY sites by Western-blotting using the site specific anti-pY antibodies. As expected, the phosphorylation levels of FAK Y576, Paxillin Y118, and p130Cas Y249 are all upregulated more or less in the active spreading stage (Fig. 5b). This observation is consistent with the slightly upregulated levels of the total pY forms of FAK, Paxillin, and p130Cas (Fig. 4b, panel II and Supplementary Table 2). It is of note that the level of FAK Y397 is not changed between the two spreading stages (Fig. 5b), explaining why the total pY level of these proteins, which is the sum of all pY sites including those upregulated, not changed, and even downregulated, are only slightly upregulated. Again, the proteins bearing the identified pY sites are highly represented by focal adhesion related pathways (Fig. 5c), corroborating the functional significance of tyrosine phosphorylation in regulating focal adhesions.

## Discussion

Cell spreading requires high coordinated actin-cytoskeleton reorganization and focal adhesion formation and turnover. Many proteins involved in the two processes including FAK, Paxillin, Cortactin, Profilin, Cofilin, and Arp2/3 components are known to be affected functionally by tyrosine phosphorylation^3^,^6^,^7^,^19^,^20^. However, how the pY level is correlated with the different spreading status has not been documented. Through morphological and immunofluorescent analyses, we separated cell spreading process into the early and the active stages. The cells at the two stages are not only different in morphology, but also in the levels of tyrosine phosphorylation. Using immunoaffinity purification of pY proteins coupled with label free quantitative proteomics, we for the first time compared the pY profiles between the two spreading stages. We found that pY levels of focal adhesion proteins, as well as of cytoskeletal proteins, were significantly increased from the early to the active spreading stages. The increased pY level implies that the structures or functions of many proteins were altered during the spreading process. Thus, our data shed a light on the future mechanistic studies addressing how the proteins are regulated to correctly play roles in the fast and dynamic changing subcellular machineries involved in cell spreading.

Attaching and spreading are two coupled processes in cell migration tightly regulated by tyrosine phosphorylation. The early spreading stage defined in the current study actually represents the firmly attached state in the other studies^5^. Dramatic tyrosine phosphorylation upon cell attachment to ECM is a typical observation due to activation of integrin signaling^5^, thus it is not surprising that many proteins were identified as pY proteins. The more interesting and valuable observation is the prolonged and increased pY level when cells are switched from the firmly attached to the active spreading stages, suggesting that extra tyrosine phosphorylation in addition to those induced by initial adhesion may be involved in regulation of cell spreading. We did not include suspended cells for the proteomic analysis because only weak pY signals can be detected when cells are in suspension^5^.

In addition to those well-known pY proteins involved in the regulation of cytoskeleton and focal adhesion, many other cytoskeletal pY proteins whose functional significance were not as frequently documented were also identified with significantly increased pY level at the active spreading stage including the components of Arp2/3 complex (Fig. 4b, panel I and Supplementary Table 2). Arp2/3 complex is the primary nucleator of new actin filaments in most migrating cells^21^, and three components ARPC2, ARPC1B, and ARPC4 were identified with increased pY level in the active spreading stage. The functional significance of tyrosine phosphorylation of these proteins has not been documented except ARPC2, of which Y202 is phosphorylated and is involved in the regulation of membrane protrusion^22^,^23^. We speculate that the increased pY level of these proteins may reflect the necessity of increasing actin filament nucleating activity to drive the membrane protrusion^3^, an essential process in cell spreading.

Proteins involved in protein translation and degradation are also highly represented by the upregulated pY proteins in the active spreading stage (Fig. 4a). This observation suggests that the dynamic spreading process may require tight protein quality control by providing fully functional newly synthesized proteins and removal of non- or mal-functional proteins. This could be achieved through regulating the activities of the respective pathways via tyrosine phosphorylation of the component proteins. Indeed, specific localization of mRNA and ribosomal proteins at adhesion sites or specialized cytoskeleton domains with a result of local protein translation have been repeatedly observed^24^,^25^,^26^. Our observation indicates that the specific localization and/or activation of proteins involved in translation is probably regulated by tyrosine phosphorylation. In contrast to cytoskeletal and focal adhesion proteins, not many pY sites were identified for such proteins (Supplementary Table 4), probably due to the low abundances of their pY forms. Specific enrichment of pY peptides instead of pY proteins for mass spectrometry analysis may be required to effectively identify the pY sites from these proteins^17^.

Supervillin and vimentin are two proteins whose pY levels were the most significantly increased and decreased, respectively, in the active spreading cells (Fig. 4b). Supervillin is an actin-binding protein that links actin filaments to the plasma membrane^27^, and is involved in the F-actin dependent integrin recycling and cell motility^28^. Supervillin also binds to myosin II and regulates its activity through MLCK during cell spreading^29^. Vimentin is the major component of the vimentin intermediate filament network. The vimentin network undergoes dramatic reorganization during cell spreading that, as a response to Rac or Cdc42 activation^30^, may be synergized with the dynamic reorganization of actin cytoskeleton. Both supervillin and vimentin are known pY proteins with a number of identified pY sites documented in the PhosphoSitePlus database. However, little information regarding the functional significance of these pY sites is available. The dramatic changes of the pY levels of the two proteins strongly indicate that their functions in cell spreading are mediated through tyrosine phosphorylation.

## Methods

### Antibodies and cell culture

The 4G10 antibody for phosphotyrosine was purchased from Millipore (Boston, MA). The antibodies for paxillin, p130Cas, and p130Cas Y249 were purchased from BD Biosciences (Franklin Lakes, NJ). The antibodies for paxillin Y31, FAK Y397, and FAK Y576/577 were purchased from Abcam (Cambridge, UK). The antibodies for paxillin Y118 was purchased from Cell Signing Technology (Boston, MA) and FAK from Proteintech (Rosemont, IL). Alexa Flour 546 phalloidin and Alexa Fluor 488 goat anti-mouse IgG were purchased from Life Technologies (New York, USA). HRP Conjugated goat anti-rabbit IgG, and HRP Conjugated goat anti-mouse IgG were purchased from CW Biotech (Beijing, China).

Human umbilical vein endothelial cell line ECV-304 was maintained in DMEM (Gibco, USA) supplemented with 10% fetal bovine serum (FBS) and 1% penicillin/streptomycin at 37 °C in a humidified atmosphere of 5% CO_2_.

### Immunofluorescence imaging

ECV-304 cells were attached to 5 μg/ml Fibronectin coated cover glass and allowed to spread for 15 min, 30 min, and 60 min at 37 °C. The cells were then fixed with 4% paraformaldehyde in PBS (pH 7.4) for 10 min at RT, and permeablized for 10 min with 0.1% Triton X-100 in PBS. Immunofluorescent staining was performed as previously described^5^, and visualized with a Leica microscope (DMI6000B, Germany). The images were analyzed with the MetaMorph software.

### Cell adhesion assay

The cell adhesion assay was performed by seeding equal number of cells on 5 μg/ml fibronectin coated cover glass. The cells were allowed to attach and spread for 15, 30, 60, 120 min, and then fixed, stained with DAPI, and imaged with fluorescence microscopy. The average number of attached cells at each time point was calculated by counting the DAPI stained nuclei in ten independent imaged fields. The average number of attached cells at the time point 120 min was used as the control to calculate the percentage of attached cells in the other time points, because there were few floating cells observed at this time point and the proportion of attached cells was considered as 100%. The proportion of floating cells at each time point was calculated by minoring the proportion of attached cells from 100%.

### Immunoaffinity purification of pY proteins

Precleared ECV-304 WCL (800 μg) in RIPA buffer (50 mM Tris-HCl, 150 mM NaCl, 1 mM EGTA, 1 mM EDTA, 1% NP40, 1% Sodium deoxycholate, 0.2% SDS) were incubated with specific anti-pY antibody (1:100) overnight at 4 °C followed by incubation for 2 h with Protein-A Sepharose beads at room temperature. The bound pY proteins were released from the protein A Sepharose beads by boiling with SDS-PAGE loading buffer. The pY proteins were then separated by SDS-PAGE and the electrophoresis was stopped when the loading dye arrived at the middle of the gel.

### In-gel digestion

The SDS-PAGE gel containing separated pY proteins was stained with Colloidal brilliant Coomassie blue. The dominant bands containing mainly the heavy chain of IgG were cut off and discarded, and this divided each lane into two major slices, with one above and the other below the heavy chain of IgG. Each gel slice was further cut into 2–3 mm^2^ pieces and destained in 25 mM ammonium bicarbonate/50% acetonitrile buffer. The samples were incubated with 10 mM DTT at 56 °C for 1 h and 55 mM iodoacetamide in the dark for 45 min, and then digested with sequencing grade trypsin (Promega, Madison, WI) in 25 mM ammonium bicarbonate at 37 °C overnight. The tryptic peptides were extracted from the gel with a buffer containing 5% trifluoroacetic acid and 50% acetonitirile. The liquid was freeze-dried with SpeedVac, and the peptides were resolubilised in 0.1% formic acid and filtered with 0.45 μm centrifugal filters.

### Mass spectrometry

The peptides were analyzed by LTQ Orbitrap Elite mass spectrometer (Thermo Fisher Scientific) coupled online to an Easy-nLC 1000 (Thermo Fisher Scientific) in the data-dependent mode. Briefly, 2 μl peptides were separated by reverse phase LC with a 75 μm (inner diameter) ×150 mm (length) analytical column packed with C18 particles of 5 μm diameter. The mobile phases for the LC contains buffer A (2% ACN, 0.1% FA) and buffer B (98% ACN, 0.1% FA). A non-linear 90 min gradient of buffer B (consisting of 3–8% for 10 min, 8–20% for 60 min, 20–30% for 8 min, 30–100% for 2 min, and 100% for 10 min) was used for the separation. All MS measurements were performed in the positive ion mode and acquired across the mass range of 300–1800 m/z. Precursor ions were measured in the Orbitrap analyzer at 240,000 resolution (at 400 m/z) and a target value of 10^6^ ions. The 20 most intense ions from each MS scan were isolated and fragmented. The CID normalized collision energy was set to 35. The raw MS data files were deposited to the ProteomeXchange Consortium (http://proteomecentral.proteomexchange.org) via the PRIDE partner repository with the dataset identifier PXD004340^31^.

### Data analysis

The database search was performed in the MaxQuant environment (version 1.5.3.30)^32^. MS/MS spectra were searched using Andromeda against the decoy UniProt human proteome database, which contains forward and reverse sequences and is concatenated with 262 frequently observed contaminants including human keratins, bovine serum proteins, and proteases. The mass tolerances for the database search were set at 4.5 ppm and 0.5 Da for precursor ions and fragment ions, respectively. Methionine oxidation, N-terminal acetylation, and phosphorylation on serine, threonine, and tyrosine residues were included as the variable modifications. Cysteine carbamidomethylation was included as the fixed modification. The maximal number of miscleavages was set at 2 and the minimum peptide length was set at 7 amino acids. The false discovery rates for both peptide and protein identifications were set at 1%.

The label-free quantitation was performed using the software MaxQuant. Briefly, the total MS intensity of each protein was quantified by the algorithm MaxLFQ^33^. The pY level of the protein FAK in each sample was determined by pY immunoprecipitation coupled with Western-blotting, and then used to further normalize the MaxLFQ quantitation result. This will reduce the bias introduced from the variation of the amount of samples injected for MS analysis. The proteins with significantly changed pY level between the two spreading stages were determined by both the Student’s *t*-test (*p* < 0.05) and fold change (>1.5).

### Bioinformatic analysis

All bioinformatic and statistical analyses were performed using Perseus (version 1.5.2.6) except the IPA analysis, which was performed using the Ingenuity Pathway Analysis software and Ingenuity Knowledge Database as previously described^4^.

## Additional Information

**How to cite this article**: Xie, Y. *et al*. Quantitative profiling of spreading-coupled protein tyrosine phosphorylation in migratory cells. *Sci. Rep.* **6**, 31811; doi: 10.1038/srep31811 (2016).

## Supplementary Material

Supplementary Table S1

Supplementary Table S2

Supplementary Table S3

Supplementary Table S4

## References

[b1] Mostafavi-Pour Z. . Integrin-specific signaling pathways controlling focal adhesion formation and cell migration. The Journal of cell biology 161, 155–167, 10.1083/jcb.200210176 (2003). 12695503 PMC2172880

[b2] Heasman S. J. & Ridley A. J. Mammalian Rho GTPases: new insights into their functions from *in vivo* studies. Nature reviews. Molecular cell biology 9, 690–701, 10.1038/nrm2476 (2008). 18719708

[b3] Rotty J. D., Wu C. & Bear J. E. New insights into the regulation and cellular functions of the ARP2/3 complex. Nature reviews. Molecular cell biology 14, 7–12, 10.1038/nrm3492 (2013). 23212475

[b4] Wang Y. . Profiling signaling polarity in chemotactic cells. Proceedings of the National Academy of Sciences of the United States of America 104, 8328–8333, 10.1073/pnas.0701103104 (2007). 17494752 PMC1895949

[b5] Wang Y. . Pseudopodium-enriched atypical kinase 1 regulates the cytoskeleton and cancer progression [corrected]. Proceedings of the National Academy of Sciences of the United States of America 107, 10920–10925, 10.1073/pnas.0914776107 (2010). 20534451 PMC2890752

[b6] Mitra S. K. & Schlaepfer D. D. Integrin-regulated FAK-Src signaling in normal and cancer cells. Curr Opin Cell Biol 18, 516–523, 10.1016/j.ceb.2006.08.011 (2006). 16919435

[b7] Webb D. J. . FAK-Src signalling through paxillin, ERK and MLCK regulates adhesion disassembly. Nat Cell Biol 6, 154–161, 10.1038/ncb1094 (2004). 14743221

[b8] Tsubouchi A. . Localized suppression of RhoA activity by Tyr31/118-phosphorylated paxillin in cell adhesion and migration. The Journal of cell biology 159, 673–683, 10.1083/jcb.200202117 (2002). 12446743 PMC2173105

[b9] Klemke R. L. . CAS/Crk coupling serves as a “molecular switch” for induction of cell migration. The Journal of cell biology 140, 961–972 (1998). 9472046 10.1083/jcb.140.4.961 PMC2141747

[b10] Chen Y. . Combined integrin phosphoproteomic analyses and small interfering RNA–based functional screening identify key regulators for cancer cell adhesion and migration. Cancer Res 69, 3713–3720, 10.1158/0008-5472.CAN-08-2515 (2009). 19351860 PMC2669841

[b11] Robertson J. . Defining the phospho-adhesome through the phosphoproteomic analysis of integrin signalling. Nat Commun 6, 6265, 10.1038/ncomms7265 (2015). 25677187 PMC4338609

[b12] Schiller H. B. . beta1- and alphav-class integrins cooperate to regulate myosin II during rigidity sensing of fibronectin-based microenvironments. Nat Cell Biol 15, 625–636, 10.1038/ncb2747 (2013). 23708002

[b13] Mann M. . Analysis of protein phosphorylation using mass spectrometry: deciphering the phosphoproteome. Trends Biotechnol 20, 261–268 (2002). 12007495 10.1016/s0167-7799(02)01944-3

[b14] Hughes S. E. Functional characterization of the spontaneously transformed human umbilical vein endothelial cell line ECV304: use in an *in vitro* model of angiogenesis. Exp Cell Res 225, 171–185, 10.1006/excr.1996.0168 (1996). 8635510

[b15] Geiger T. & Zaidel-Bar R. Opening the floodgates: proteomics and the integrin adhesome. Curr Opin Cell Biol 24, 562–568, 10.1016/j.ceb.2012.05.004 (2012). 22728062

[b16] Hornbeck P. V. . PhosphoSitePlus: a comprehensive resource for investigating the structure and function of experimentally determined post-translational modifications in man and mouse. Nucleic Acids Res 40, D261–270, 10.1093/nar/gkr1122 (2012). 22135298 PMC3245126

[b17] Rush J. . Immunoaffinity profiling of tyrosine phosphorylation in cancer cells. Nature biotechnology 23, 94–101, 10.1038/nbt1046 (2005). 15592455

[b18] Calalb M. B., Polte T. R. & Hanks S. K. Tyrosine phosphorylation of focal adhesion kinase at sites in the catalytic domain regulates kinase activity: a role for Src family kinases. Mol Cell Biol 15, 954–963 (1995). 7529876 10.1128/mcb.15.2.954 PMC231984

[b19] Martinez-Quiles N., Ho H. Y., Kirschner M. W., Ramesh N. & Geha R. S. Erk/Src phosphorylation of cortactin acts as a switch on-switch off mechanism that controls its ability to activate N-WASP. Mol Cell Biol 24, 5269–5280, 10.1128/MCB.24.12.5269-5280.2004 (2004). 15169891 PMC419870

[b20] Bravo-Cordero J. J., Magalhaes M. A., Eddy R. J., Hodgson L. & Condeelis J. Functions of cofilin in cell locomotion and invasion. Nature reviews. Molecular cell biology 14, 405–415, 10.1038/nrm3609 (2013). 23778968 PMC3878614

[b21] Arber S. . Regulation of actin dynamics through phosphorylation of cofilin by LIM-kinase. Nature 393, 805–809 (1998). 9655397 10.1038/31729

[b22] Choi C. H., Thomason P. A., Zaki M., Insall R. H. & Barber D. L. Phosphorylation of actin-related protein 2 (Arp2) is required for normal development and cAMP chemotaxis in Dictyostelium. The Journal of biological chemistry 288, 2464–2474, 10.1074/jbc.M112.435313 (2013). 23223240 PMC3554915

[b23] LeClaire L. L. 3rd, Baumgartner M., Iwasa J. H., Mullins R. D. & Barber D. L. Phosphorylation of the Arp2/3 complex is necessary to nucleate actin filaments. The Journal of cell biology 182, 647–654, 10.1083/jcb.200802145 (2008). 18725535 PMC2518704

[b24] Chicurel M. E., Singer R. H., Meyer C. J. & Ingber D. E. Integrin binding and mechanical tension induce movement of mRNA and ribosomes to focal adhesions. Nature 392, 730–733, 10.1038/33719 (1998). 9565036

[b25] Humphries J. D., Paul N. R., Humphries M. J. & Morgan M. R. Emerging properties of adhesion complexes: what are they and what do they do? Trends Cell Biol 25, 388–397, 10.1016/j.tcb.2015.02.008 (2015). 25824971

[b26] Katz Z. B. . Mapping translation ‘hot-spots’ in live cells by tracking single molecules of mRNA and ribosomes. Elife 5, 10.7554/eLife.10415 (2016). PMC4764586 26760529

[b27] Pestonjamasp K. N., Pope R. K., Wulfkuhle J. D. & Luna E. J. Supervillin (p205): A novel membrane-associated, F-actin-binding protein in the villin/gelsolin superfamily. The Journal of cell biology 139, 1255–1269 (1997). 9382871 10.1083/jcb.139.5.1255 PMC2140202

[b28] Fang Z. . The membrane-associated protein, supervillin, accelerates F-actin-dependent rapid integrin recycling and cell motility. Traffic 11, 782–799, 10.1111/j.1600-0854.2010.01062.x (2010). 20331534 PMC2888608

[b29] Takizawa N., Ikebe R., Ikebe M. & Luna E. J. Supervillin slows cell spreading by facilitating myosin II activation at the cell periphery. Journal of cell science 120, 3792–3803, 10.1242/jcs.008219 (2007). 17925381

[b30] Meriane M. . Cdc42Hs and Rac1 GTPases induce the collapse of the vimentin intermediate filament network. The Journal of biological chemistry 275, 33046–33052, 10.1074/jbc.M001566200 (2000). 10900195

[b31] Vizcaino J. A. . 2016 update of the PRIDE database and its related tools. Nucleic Acids Res 44, D447–456, 10.1093/nar/gkv1145 (2016). 26527722 PMC4702828

[b32] Cox J. & Mann M. MaxQuant enables high peptide identification rates, individualized p.p.b.-range mass accuracies and proteome-wide protein quantification. Nature biotechnology 26, 1367–1372, 10.1038/nbt.1511 (2008). 19029910

[b33] Cox J. . Accurate proteome-wide label-free quantification by delayed normalization and maximal peptide ratio extraction, termed MaxLFQ. Mol Cell Proteomics 13, 2513–2526, 10.1074/mcp.M113.031591 (2014). 24942700 PMC4159666

